# Under-diagnosis of vector-borne diseases among individuals suspected of having Scrub Typhus in South Korea

**DOI:** 10.1371/journal.pone.0286631

**Published:** 2023-06-02

**Authors:** Eun Jeong Won, Seong Hoon Kim, Kyeong Hwan Byeon, Chae-Hyeon Jeon, Seung-Ji Kang, Joo-Heon Park, Seung-Jung Kee, Hyun-Woo Choi

**Affiliations:** 1 Departments of Parasitology and Tropical Medicine, Chonnam National University Medical School, Hwasun, South Korea; 2 Department of Laboratory Medicine, Asan Medical Center, University of Ulsan College of Medicine, Seoul, South Korea; 3 Department of Laboratory Medicine, Chonnam National University Hospital, Gwangju, South Korea; 4 Department of Infectious Disease, Chonnam National University Bitgoeul Hospital, Gwangju, South Korea; 5 Department of Laboratory Medicine, Chonnam National University Bitgoeul Hospital, Gwangju, South Korea; University of Bari, ITALY

## Abstract

Due to environmental and ecological changes and suitable habitats, the occurrence of vector-borne diseases is increasing. We investigated the seroprevalence of four major vector-borne pathogens in human patients with febrile illness who were clinically suspected of having Scrub Typhus (ST) caused by *Orientia tsutsugamushi*. A total of 187 samples (182 patient whole blood and sera samples, including 5 follow-up) were collected. Antibodies to *Anaplasma phagocytophilum*, *Ehrlichia chaffeensis*, *Borrelia burgdorferi*, and *Bartonella henselae* were tested by using indirect immunofluorescence assays. Molecular diagnoses were performed using real-time PCR. Of the 182 cases, 37 (20.3%) cases were designated as confirmed cases of ST, and the remaining 145 (79.7%) cases as other febrile diseases (OFDs). The seroprevalence of *A*. *phagocytophilum*, *E*. *chaffeensis*, *B*. *burgdorferi*, and *B*. *henselae* was 51.4% (19/37), 10.8% (4/37), 86.5% (32/37), and 10.8% (4/37) among the ST group, and 42.8% (62/145), 10.4% (19/145), 57.7% (105/145), and 15.9% (29/145) among the OFD group, respectively. There were no significant differences in the seroprevalence between the ST and the OFD groups. Considering the co-occurrence, 89.0% (162/182) had at least one antibody to tick-borne pathogens, 37.0% (60/162) were positive for two pathogens, 17.3% (28/162) for three pathogens, and 6.2% (10/162) for four pathogens. In real-time PCR, *O*. *tsutsugamushi* was positive in 16 cases [15 (40.5%) in ST group and 1 (2.2%) in OFD group], and the four other pathogens were negative in all cases except one confirmed as anaplasmosis. In evaluating the five follow-up samples, the appearance of new antibodies or an increase in the pre-existing antibody titers was detected. Our data highlighted that acute febrile illness and manifestations suggestive of a vector-borne infection must be recognized and further considered for coinfections in clinical practice and the laboratory.

## Introduction

Global warming, environmental and ecological changes, and suitable habitats have increased the impact that ticks and mites have on humans, and are associated with the frequent emergence or re-emergence of tick- or arthropod-borne diseases with zoonotic characteristics [1, 2]. The growing number of such vector-mediated infection cases, and in particular, fatal viral epidemics in humans, has recently increased the extent of public awareness [2]. The “One Health” initiative of the World Health Organization (WHO) also encourages the development of strategies inhibiting and controlling vector-borne infections in humans and animals.

Vectors such as ticks, fleas, mites, or mosquitos can transmit bacterial, parasitic, and viral pathogens, and such vectors often host more than one agent simultaneously. Rickettsiales (genera *Anaplasma*, *Ehrlichia*, and *Rickettsia*), *Bartonella*, and *Borrelia* are the most common vector-borne pathogens [3]. *Anaplasma phagocytophilum* is an emerging, Gram-negative, obligate intracellular bacteria transmitted by *Ixodes* ticks [4]. In humans, infection ranges from asymptomatic to severe disease that can present with pancytopenia, multi-organ failure, or death. In addition, *Ehrlichia chaffeensis* also causes the life-threatening disease called Human Monocytic Ehrlichiosis, with acute sepsis and toxic shock-like symptoms that can evolve into multi-organ failure or death [5, 6]. Early clinical and laboratory diagnoses are problematic due to non-specific flu-like symptoms and limitations in the current diagnostic testing [5, 7]. Lyme disease caused by pathogenic members of the *Borrelia burgdorferi* s.l. complex, typically begins with erythema migrans (˜80%), but ∼18% of patients have non-specific symptoms such as malaise, fatigue, headache, arthralgias, myalgias, fever, and regional lymphadenopathy without recognition of erythema migrans, for which differential diagnoses are required [8]. Likewise, infection of *Bartonella henselae*, which is a gram-negative, coccobacillus, facultative intracellular bacterium, also manifests diverse and nonspecific symptoms above mentioned, which could initially lead to misdiagnosis as other diseases [9]. People usually contract the disease from cats infected with *B*. *henselae*, but flea or tick bite cases have been reported [10]. *B*. *henselae* is known to be transmitted by cat’s scratch or bite, due to the contamination of saliva and nails with the bacteria, but the other *Bartonella* spp. can be transmitted by ticks or fleas bites, such as *Bartonella birtlesii* [10–12].

Scrub Typhus (ST) is an acute febrile disease caused by *Orientia tsutsugamushi*, which is transmitted by larval-stage trombiculid mites [13]. In South Korea, ST is endemic and is one of the leading public health concerns, with infections most frequently occurring between October and November [14]. The clinical presentations of ST and other vector-borne diseases are similar at the early stage of infection: signs and symptoms typically develop within 1 and 2 weeks of infection and include fever, headache, malaise, and gastrointestinal symptoms [15]. Therefore, it is difficult to identify the causative pathogen based on the clinical presentation. Moreover, the clinical vector-borne disease spectrum ranges from asymptomatic to fatal and is disproportionately high in children and older adults who may not show distinct features [16]. Thus, a broad view and an extensive examination are essential for diagnosing and treating vector-borne diseases.

Despite the clinical relevance of vector-borne disease, in-depth epidemiological studies and research investigations are still lacking in Korea. Here, we investigate the seropositivity and DNA detection to the four major vector-borne pathogens (*Anaplasma phagocytophilum*, *Ehrlichia*, *Borrelia*, and *Bartonella henselae*) in a cohort of patients with febrile illness who were clinically suspected of having ST.

## Materials and methods

### Study cohort and sample collection

From September 2019 to June 2020, residual samples of 187 whole blood in EDTA tubes and 187 sera in serum separator tubes from 182 patients were collected after the ordered routine laboratory testing at Chonnam National University Hospital (CNUH), South Korea. Of the 187 sets of whole blood and serum, 177 were single-drawn sample sets from 177 patients, and 10 were paired sample sets consisting of 5 first-drawn and 5 randomly collected follow-up samples from 5 patients. Most presented febrile illness and were clinically suspected of having ST. Medical data of the patients, including age, gender, clinical history, symptoms, laboratory findings, and the final clinical diagnosis, were obtained through retrospective electronic medical record reviews, with personally identifiable information removed. Collected laboratory parameters were as follows: white blood cell with differential count (neutrophils, lymphocytes, monocytes, eosinophils, and basophils), hemoglobin, platelet (Sysmex XN-1000; Sysmex Corporation, Kobe, Japan); erythrocyte sedimentation rate (TEST 1 BCL; Alifax, Polverara, Italy); fibrinogen, activated partial thromboplastin time (STA-R; Diagnostica Stago, Asnieres, France); C-reactive protein, aspartate aminotransferase, alanine aminotransferase, alkaline phosphatase, lactate dehydrogenase, blood urea nitrogen, creatinine, glucose, total bilirubin, direct bilirubin (AU5800; Olympus, Tokyo, Japan) and antibody against *O*. *tsutsugamushi*. The anti-*O*.*tsutsugamushi* antibody test was performed using commercially available lateral-flow-format immunochromatographic assay kits (SD Bio-line, Youngin, Korea). The final diagnoses of ST were defined by physicians according to clinical and laboratory findings: history of outdoor activities, typical eschar or maculopapular rash, fever, therapeutic response to treatment, and anti-*O*.*tsutsugamushi* antibody test results [17].

### Indirect immunofluorescent assays for antibodies to other vector-borne pathogens

**Fig 1:**
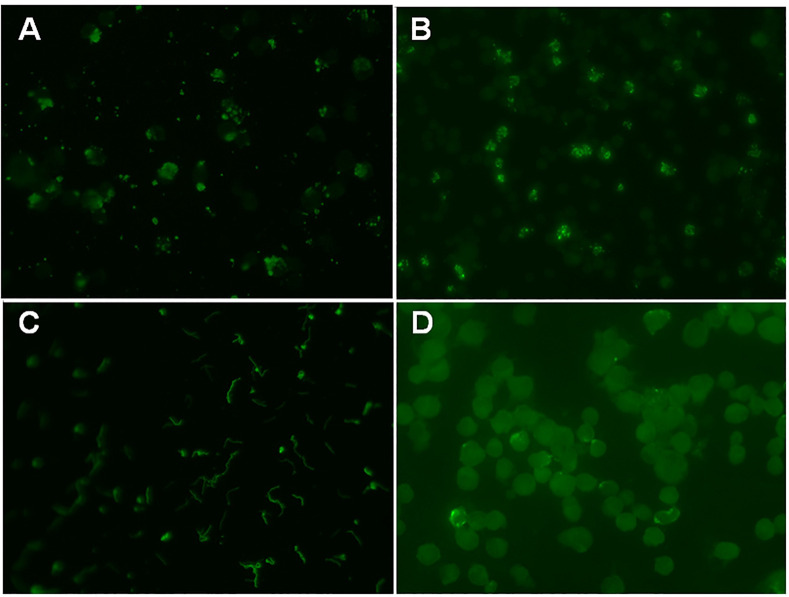
Representative images of the indirect immunofluorescence assays (IFAs) for the IgG antibody against the major vector-borne pathogens performed in this study. (A) Positive anti-*Anaplasma phagocytophilum* IgG appears as one or more distinct apple-green phagosomes (morulae) within the cytoplasm of the infected cells (IFA, × 200). (B) Positive anti-*Ehrlichia chaffeensis* IgG appears as peripheral clusters of distinct apple-green inclusion bodies within the infected erythrocytes (IFA, × 200). (C) Positive anti-*Borrelia burgdorferi* IgG appears as bright staining of characteristic spirochetes (IFA, × 200). (D) Positive anti-*Bartonella henselae* IgG appears as brightly fluorescent sharp, regularly stained coccobacilli within the cytoplasm of the fixed Vero cells (IFA, × 200).

Commercially available IFA test kits containing the positive and negative control reagents were used to analyze the immunoglobulin G (IgG) of anti-*Borrelia burgdorferi*, anti-*Anaplasma phagocytophilum*, anti-*Bartonella henselae*, and anti-*Ehrlichia chaffeensis* (Fuller Laboratories, Fullerton, CA, USA). All 187 sera were screened at a 1:64 dilution, according to the manufacturer’s instructions. We serially diluted the positive controls at ratios of 1:64, 1:128, 1:256, 1:512, and 1:1,024. The negative control and the serial dilutions of the positive control were assayed with the samples in each run. First, the samples were placed on a slide in contact with the substrate and incubated. The slide was then washed in phosphate-buffered saline to remove unbound antibodies. In the second stage, each well was overlaid with a solution of a fluorescein-labeled antibody to human IgG. The antigen–antibody complexes reacted with the anti-human IgG. Each slide was washed, dried, mounted, and interpreted under a fluorescence microscope (Fig 1). The manufacturer recommended the cutoff titer as 1:512; therefore, the fluorescence intensity of the 1:512 diluted positive control was set to the cutoff level to determine a positive test result. Samples with less fluorescence intensity than the 1:512 positive control were interpreted as negative. The fluorescence intensity of the positive samples was compared to the positive controls, and the titers were graded as follows: 1+, the intensity of the 1:512 diluted positive control; 2+, the intensity of the 1:256 positive control; 3+, the intensity of the 1:128 positive control; and 4+, the intensity of the 1:64 positive control. Titers graded as 1+ and 2+ were defined as low titers, and 3+ and 4+ were defined as high titers.

### Real-time PCR for vector-borne pathogens

DNA was extracted from all 187 whole blood samples using the QIAamp DNA Mini Kit (QIAGEN GmbH, Hilden, Germany) according to the manufacturer’s instructions. The real-time PCR was performed using commercial kits for vector-borne pathogens: the Power Chek *O*. *tsutsugamushi* Real-time PCR Kit, PowerChek *Ehrlichia/Anaplasma* Real-time PCR Kit, and PowerChek *Rickettsia/Borrelia/Bartonella* Real-time PCR Kit (Kogene Bio-tech, Seoul, South Korea). Any positive reaction was re-confirmed by additional PCR and sequencing as previously described (S1 Table) [14, 18–21].

### Statistical analysis

The results of the 182 single and first-drawn samples were included in the statistics, and the 5 follow-up samples from 5 patients were not included. Fisher’s exact test or a chi-square test was used to compare categorical variables. Student’s t-test was used to compare the continuous variables. A *P*-value of < 0.05 was considered statistically significant. All statistical analyses were performed using either SPSS 27.0 (IBM Corp., Armonk, NY, USA) or the diagnostic test evaluation calculator (MedCalc, Ostend, Belgium).

### Ethics statement

The collection of samples for this study was conducted in accordance with the guidelines and approval of the Institutional Review Board of Chonnam National University Hospital (approval no. CNUH-2020-117). A waiver of consent was granted by given the nature of the project dealing with the remaining samples, and no information was used that could lead to patient identification.

## Results

### Demographics and clinical characteristics

Overall, 37 (20.3%) cases were designated as confirmed cases of ST, and the remaining 145 (79.7%) cases were designated as OFD group (Table 1). The OFD group comprised gastrointestinal diseases (n = 33), respiratory infections (n = 24), central nervous system diseases (n = 17), allergic diseases (n = 17), heart disease (n = 9), malignancy (n = 8), leptospirosis (n = 2), hemorrhagic fever with renal syndrome (n = 2), anaplasmosis (n = 2), severe fever with thrombocytopenia syndrome virus (SFTS) (n = 1) and undifferentiated fever (n = 30). The median age of the ST group was significantly higher than the OFD group (73.1 vs. 64.0 years, *P* = 0.018), and female predominance was found in the ST group (67.6% vs. 44.8%, *P* = 0.017). In addition, fever, tick-bite history or eschar, and a history of outdoor activity were more frequent in the ST group. Differences in the laboratory parameters were not significant between the ST and OFD groups, except for in the % of lymphocytes and % of basophils.

**Table 1:** Demographics and clinical characteristics of the 182 patients enrolled in this study.

Characteristics		Scrub typhus (n = 37)	Other febrile disease (n = 145)	*P* ^*^
Age, year, median (IQR)		73.1 (61 − 79.1)	64.0 (48.1–77.1)	0.018
Female, n (%)		25 (67.6)	65 (44.8)	0.017
Clinical manifestations, n (%)				
	fever	35 (94.6)	91 (62.8)	<0.001
	headache	0 (0)	12 (8.3)	0.129
	myalgia	3 (8.1)	8 (5.5)	0.698
	neurologic manifestations	8 (21.6)	26 (17.9)	0.638
	lymphadenopathy	0 (0)	5 (3.4)	0.585
	gastrointestinal symptoms	4 (10.8)	22 (15.2)	0.606
	respiratory symptoms	5 (13.5)	23 (15.9)	1
	rash	9 (24.3)	19 (13.1)	0.123
	cardiac symptoms	2 (5.4)	7 (4.8)	1
	cytopenia	4 (10.8)	6 (4.1)	0.121
	urological symptoms	1 (2.7)	5 (3.4)	1
	nausea/vomiting	1 (2.7)	5 (3.4)	1
	tick-bite history or eschar	29 (78.4)	3 (2.1)	<0.001
	outdoor activity history	29 (78.4)	22 (15.2)	<0.001
Laboratory findings (mean ± SD)				
	WBC, 10^3/μL	7.5 ± 3.7	9 ± 6.6	0.178
	Hemoglobin, g/dL	12.1 ± 1.6	11.6 ± 2.2	0.201
	Platelet, 10^3/μL	161.8 ± 82.1	194 ± 138.5	0.177
	Neutrophil, %	66 ± 16.7	70.9 ± 18.1	0.141
	Lymphocyte, %	25.5 ± 15.1	18.7 ± 13.9	0.010
	Monocyte, %	7.4 ± 3.3	7.4 ± 4.7	0.949
	Eosinophil, %	0.4 ± 0.7	2.3 ± 6.8	0.089
	Basophil, %	0.7 ± 0.5	0.4 ± 0.5	0.001
	CRP, mg/dL	8.3 ± 7.1	9.6 ± 9.9	0.463
	ESR, mm	32.8 ± 16.5	36.3 ± 29.2	0.746
	AST, U/L	115.6 ± 166.2	222 ± 725.3	0.384
	ALT, U/L	87.1 ± 121.5	154.5 ± 492.4	0.417
	ALP, U/L	146.6 ± 101.4	129.9 ± 106.5	0.429
	LDH, U/L	853.6 ± 375.1	1529.1 ± 4590.5	0.448
	BUN, mg/dL	19.9 ± 13.5	23.9 ± 20.4	0.274
	Creatinine, mg/dL	0.9 ± 0.6	1.3 ± 1.5	0.075
	Glucose, mg/dL	160.7 ± 95.7	158 ± 84.5	0.888
	Fibrinogen, mg/dL	315.1 ± 113.7	423.5 ± 184.5	0.055
	Total bilirubin, mg/dL	0.9 ± 0.8	1.3 ± 2	0.204
	Direct bilirubin, mg/dL	0.7 ± 0.9	1.1 ± 1.8	0.423
	aPTT, second	33 ± 7	35.4 ± 11	0.271

*Age and laboratory findings were analyzed by Fisher’s exact test or Chi-square test; sex and clinical manifestations by Student’s t-test. *P* < 0.05 were considered statistically significant.

Abbreviations: AST, aspartate aminotransferase; ALP, alkaline phosphatase; ALT, alanine aminotransferase; aPTT, activated partial thromboplastin time; BUN, blood urea nitrogen; CRP, C-reactive protein; ESR, erythrocyte sedimentation rate; IQR, interquartile range; LDH, lactate dehydrogenase; n, number; SD, standard deviation; WBC, whole blood cell.

### Seroprevalence and titers of antibodies to vector-borne pathogens

The overall seroprevalence of antibodies to *B*. *burgdorferi* was 75.3%; for *A*. *phagocytophilum*, 44.5%; for *B*. *henselae*, 18.1%; and for *E*. *chaffeensis*, 12.6% (Table 2). Among the ST group, the seroprevalence to *A*. *phagocytophilum*, *E*. *chaffeensis*, *B*. *burgdorferi*, and *B*. *henselae* was 51.4%, 10.8%, 86.5%, and 10.8%, respectively. Among the OFD group, the seroprevalence to *A*. *phagocytophilum*, *E*. *chaffeensis*, *B*. *burgdorferi*, and *B*. *henselae* was 42.8%, 10.4%, 57.7%, and 15.9%, respectively. There was no significant difference in the seropositivity to *A*. *phagocytophilum*, *E*. *chaffeensis*, *B*. *burgdorferi*, and *B*. *henselae* between the ST and the OFD groups. As for the antibody titers, the ST group more frequently exhibited high titers of *A*. *phagocytophilum* and *B*. *burgdorferi*, but a low titer of *E*. *chaffeensis*. Low titers of *A*. *phagocytophilum*, *E*. *chaffeensis*, and *B*. *henselae* were more frequently observed among the OFD group. The proportion of cases with *B*. *burgdorferi* having the highest titer (4+) was significantly higher in the ST group than in the OFD group (43.2% vs. 19.2%, *P* = 0.02).

**Table 2:** Seroprevalence and titers of antibodies to *Anaplasma phagocytophilum*, *Ehrlichia chaffeensis*, *Borrelia burgdorferi*, and *Bartonella henselae* in Scrub Typhus-confirmed cases and other febrile disease groups in this study.

		Seroprevalence of antibodies, n (%)		
Targets and titers		Scrub Typhus (n = 37)	Other febrile disease (n = 145)	Total (n = 182)
*Anaplasma phagocytophilum*		19 (51.4)	62 (42.8)	81 (44.5)
	4+	5 (13.5)	11 (6)	16 (8.8)
	3+	5 (13.5)	17 (9.3)	22 (12.1)
	2+	6 (16.2)	16 (8.8)	22 (12.1)
	1+	3 (8.1)	18 (9.9)	21 (11.5)
*Ehrlichia chaffeensis*		4 (10.8)	19 (10.4)	23 (12.6)
	4+	0 (0)	1 (0.5)	1 (0.5)
	3+	1 (2.7)	3 (1.6)	4 (2.2)
	2+	1 (2.7)	7 (3.8)	8 (4.4)
	1+	2 (5.4)	8 (4.4)	10 (5.5)
*Borrelia burgdorferi*		32 (86.5)	105 (57.7)	137 (75.3)
	4+	16 (43.2)^†^	35 (19.2) ^†^	51 (28)
	3+	6 (16.2)	28 (15.4)	34 (18.7)
	2+	8 (21.6)	28 (15.4)	36 (19.8)
	1+	2 (5.4)	14 (7.7)	16 (8.8)
*Bartonella henselae*		4 (10.8)	29 (15.9)	33 (18.1)
	4+	1 (2.7)	4 (2.2)	5 (2.7)
	3+	1 (2.7)	4 (2.2)	5 (2.7)
	2+	1 (2.7)	12 (6.6)	13 (7.1)
	1+	1 (2.7)	9 (4.9)	10 (5.5)

All were negative to real-time PCR assay for the four vector-borne pathogens.

†*P* = 0.020. The difference in the proportions between scrub typhus-confirmed cases and other febrile disease groups was calculated by Fisher’s exact test or Chi-square test. *P* < 0.05 were considered statistically significant. All other *P*-values were > 0.05.

### Co-occurrence rate of antibodies to vector-borne pathogens

**Fig 2:**
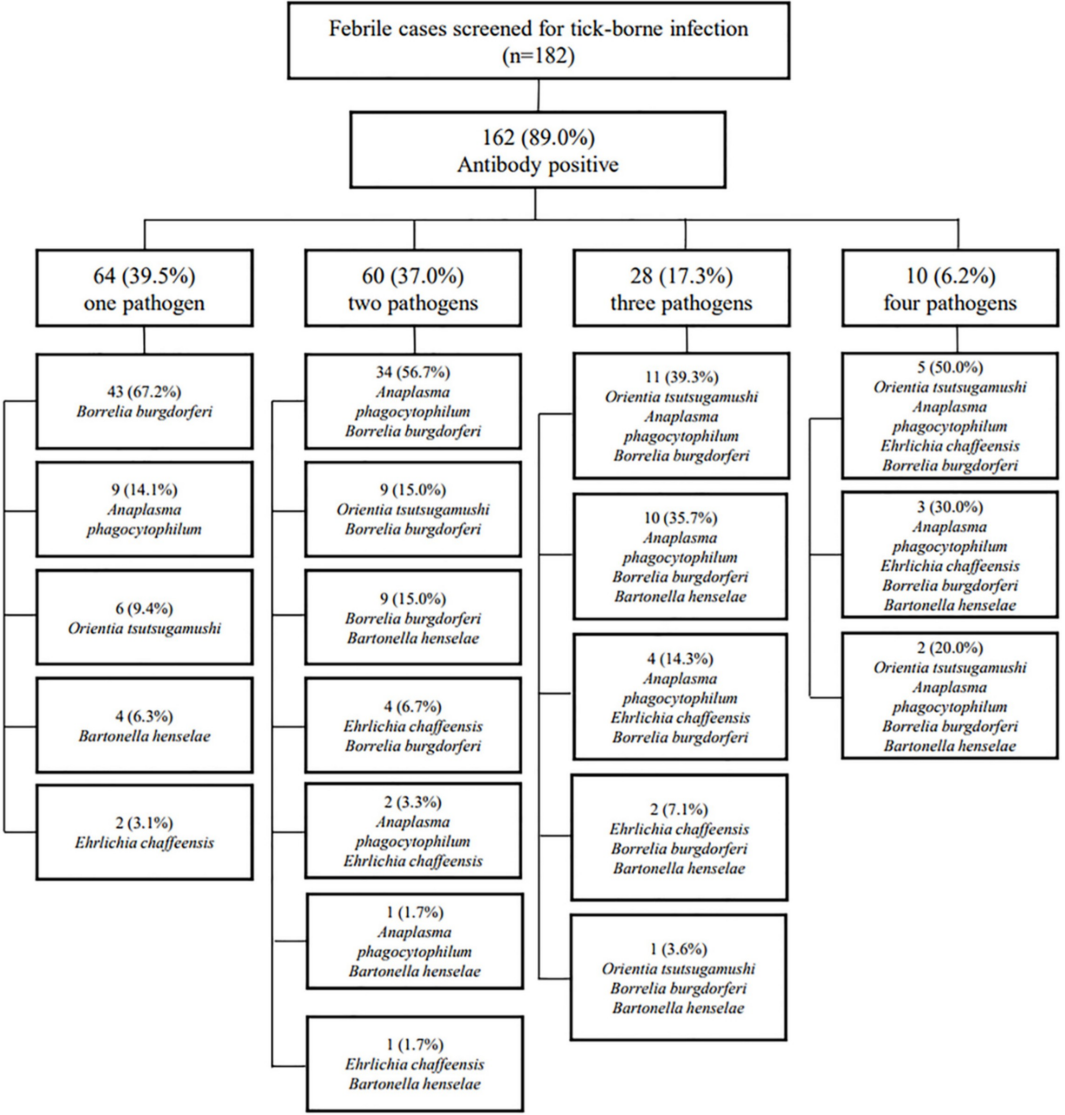
A schematic diagram presenting the seropositive rate and co-occurring pathogens of the 182 cases with febrile illness screened for vector-borne infection in this study. The antibodies to *Anaplasma phagocytophilum*, *Ehrlichia chaffeensis*, *Borrelia burgdorferi*, and *Bartonella henselae* were tested with commercial IFA assay kits (Fuller Laboratories, Fullerton, CA, USA). Abbreviations: n—number.

Fig 2 displays the overall co-occurrence rate and the co-occurring pathogens of the 182 cases with febrile illness screened for vector-borne infection in this study, and 89.0% (162/182) of them harbored at least one antibody to vector-borne pathogens. Among them, 39.5% (64/162) were antibody positive for one pathogen, 37.0% (60/162) were positive for two pathogens, 17.3% (28/162) for three pathogens, 6.2% (10/162) for four pathogens, and none for five pathogens. *B*. *burgdorferi* accounted for 67.2% (43/64) of the single antibody-positive group, followed by *A*. *phagocytophilum* at 14.1% (9/64). Those two pathogens were also the most common co-occurring pathogens in the double-antibody-positive group at 56.7% (34/60). The simultaneous incidence of *A*. *phagocytophilum* and *B*. *burgdorferi* were the highest among the triple antibody-positive group for 89.3% (25/28), frequently accompanied by *O*. *tsutsugamushi* or *B*. *henselae*. The co-occurrence of *O*. *tsutsugamushi*, *A*. *phagocytophilum*, and *B*. *burgdorferi* was observed in 70.0% of the quadruple antibody-positive groups. Additionally, the co-occurrence rate of each antibody among the 60.5% (98/162) cases seropositive for multiple pathogens was accessed and is shown in Table 3.

**Table 3:** The co-occurrence rate of each antibody among the 98 cases positive for multiple (two to four) pathogens.

Co-occurrence, n (%)	*Orientia tsutsugamushi*	*Anaplasma phagocytophilum*	*Ehrlichia chaffeensis*	*Borrelia burgdorferi*	*Bartonella henselae*
*Orientia tsutsugamushi* (n = 29)		18 (62.1)	5 (17.2)	28 (96.6)	3 (10.3)
*Anaplasma phagocytophilum* (n = 73)	18 (24.7)		14 (19.2)	69 (94.5)	16 (21.9)
*Ehrlichia chaffeensis* (n = 21)	5 (23.8)	14 (66.7)		18 (85.7)	6 (28.6)
*Borrelia burgdorferi* (n = 98)	28 (28.6)	69 (70.4)	18 (18.4)		27 (27.6)
*Bartonella henselae* (n = 29)	3 (10.3)	16 (55.2)	6 (20.7)	27 (93.1)	

### Real-time PCR for vector-borne pathogens

The real-time PCR for *O*. *tsutsugamushi* was positive in 16 cases; 12 were antibody-positive cases, and 4 were antibody-negative cases. Real-time PCR assays for *E*. *chaffeensis*, *Borrelia burgdorferi*, *B*. *henselae* were all negative, but only one case was positive. This positive reaction for *A*. *phagocytophilum* was confirmed by additional sequencing, and the sequence was deposited to GenBank (Accession no. OQ750553) (S1 Fig). This positive sample was derived from the patient who was clinically diagnosed with anaplasmosis by morulae found within granulocytes in the peripheral blood smear (PBS).

### Paired follow-up sample data

In addition, we further investigated the paired follow-up samples from five patients (Table 4). They were all clinically diagnosed with ST. In case 1, antibodies for *A*. *phagocytophilum* and *B*. *burgdorferi* appeared on day 18, which were negative on day 0. In case 2, antibodies for *O*. *tsutsugamushi* and *A*. *phagocytophilum* appeared. The increase in titer was also observed in case 3. In case 4, the antibodies for *O*. *tsutsugamushi*, *A*. *phagocytophilum*, and *B*. *henselae* turned positive on day 10. In case 5, the antibody for *E*. *chaffeensis* appeared on day 2. The follow-up samples demonstrated new antibody appearance or some titer changes, but there were no cases of negative changes in existing antibodies.

**Table 4:** The clinical and serological data of the follow-up samples from five patients.

Case	Age/Sex	Clinical diagnosis	Symptoms	Tick bite history or eschar	Sample ID	Sample collection day	Antibody, titer^†^				
							*Orientia tsutstugamushi*	*Anaplasma phagocytophilum*	*Ehrlichia chaffeensis*	*Borrelia burgdorferi*	*Bartonella henselae*
1	78/F	ST	fever, skin rash	yes	039	day 0^*^	positive	negative	negative	negative	negative
					070	day 18	positive	positive, 4+	negative	positive, 3+	negative
2	60/F	ST	fever, vomiting	yes	047	day 0	negative	negative	negative	positive, 2+	negative
					055	day 8	positive	positive, 2+	negative	positive, 3+	negative
3	80/M	ST	fever	yes	059	day 0	positive	positive, 2+	negative	positive, 2+	negative
					089	day 10	positive	positive, 2+	negative	positive, 4+	negative
4	77/M	ST	fever, skin rash	yes	101	day 0	negative	negative	negative	positive, 4+	negative
					155	day 10	positive	positive, 2+	negative	positive, 3+	positive, 3+
5	85/M	ST	fever, general weakness	yes	099	day 0	positive	positive, 3+	negative	positive, 4+	negative
					106	day 2	positive	positive, 3+	positive, 2+	positive, 4+	negative

^*^day 0 is the day on which the first sample from the patient was obtained.

^†^ Antibody tests for *O*. *tsutsugamushi* were performed by immunochromatographic assay and for the other four pathogens by indirect immunofluorescent assay.

Abbreviations: F, female; ID, identification number; IFA, immunofluorescence assay; M, male; ST, Scrub Typhus.

## Discussion

Tick- and arthropod-borne diseases in humans often share similar clinical features but are epidemiologically and etiologically distinct. In South Korea, it is estimated that more than 10,000 patients are treated every year for tick- or arthropod-borne diseases, such as ST or spotted fever. Despite the clinical relevance of vector-borne infections, in-depth epidemiological studies and research investigations are lacking in Korea. The antibodies to several vector-borne pathogens may overlap because trombiculid mites and ticks share many of the same habitats, and even a single elevated antibody titer can be evidence of exposure to a pathogen, although not sufficient to confirm acute infection. In this study, we demonstrated that many ST patients have the possibility of co-exposure to multiple vector-borne pathogens.

Ticks can transmit both the *Borrelia* Lyme group and *Borrelia* relapsing fever group, including the *Borrelia* hard tick-borne relapsing fever group *(Borrelia miyamotoi*) [22]. The *B*. *burgdorferi* sensu lato (s.l.) complex is a diverse group of worldwide-distributed bacteria, comprised of 21 different species [8]. Eleven species from the *B*. *burgdorferi* s.l. complex were identified in and strictly associated with Eurasia and, until now, only *B*. *afzelii*, *B*. *garinii* and *B*. *valaisiana* have been reported in Korea [23–25]. Lyme disease, caused by pathogenic members of the *B*. *burgdorferi* s.l. complex, typically begins with erythema migrans (˜80%), but ∼18% of patients have non-specific symptoms such as malaise, fatigue, headache, arthralgias, myalgias, fever, and regional lymphadenopathy without recognition of erythema migrans, for which differential diagnoses are required [26]. Recent Korean data reported 8.1% seroprevalence to *B*. *burgdorferi* antibodies among forestry workers in National Park Offices, and they used an in-house IFA method [27]. At the present study, commercially available IFA kits were used, and the high seropositive rate of *B*. *burgdorferi* was noticed in both ST and OFD groups, in addition to high titers. Interpreting a single positive serologic result as an infection is challenging, especially in endemic areas [28]. However, in non-endemic areas with low seropositivity, like South Korea, a single elevated antibody titer can support the possibility of infection with vector-borne pathogens, especially for those with suspicious clinical manifestations and history. Therefore, an additional serologic evaluation for *B*. *burgdorferi* antibody might help identify the infection in Korea.

It should also be noted that the co-occurrence of *B*. *burgdorferi* with *A*. *phagocytophilum* or *O*. *tsutsugamushi* antibodies was the most frequently found in this study. Several ecological factors could be considered for these serological results. *O*. *tsutsugamushi* is transmitted through bites of trombiculid mites, but not through ticks’ bites. However, the co-occurrence of antibodies to several vector-borne bacteria could be predicted since trombiculid mites and ticks transmit *B*. *burgdorferi* or *Anaplasma* spp. and share many of the same habitats [29]. In a previous report, *A*. *phagocytophilum* was detected both in *Ixodes persulcatus* ticks and the blood of humans after tick bites [30]. In addition, several researchers previously announced that *Rickettsiae* could be transmitted by *Ixodes* sp. *(Rickettsia helvetica* and *R*. *monacensis*), *Haemaphysalis longicornis* and *Dermacentor marginatus* ticks (*Rickettsia raoultii*), or other ticks [31, 32]. Regarding climate change, it is known that warming impacts the activity and aggressiveness of ticks, causing human attacks and the possibility of transmission of severe tick-borne pathogens to increase [33]; thus, further caution to infection of various VBDs should be taken as concerns grow in Korea.

In addition to *Borrelia*, both *A*. *phagocytophilum* and *E*. *chaffeensis* were found in *H*. *longicornis* and *I*. *persulcatus* ticks throughout Korea [34]. Although several seroepidemiological and molecular studies have shown that these agents are present in Asia [35, 36], suspecting and diagnosing those infections is not easy. Previous seroprevalence studies showed that 1.8% of serum samples from patients with acute fever were positive for *A*. *phagocytophilum* through IFA testing [35]. In 2003, the first Korean case of *A*. *phagocytophilum* was detected using molecular and serological methods in Chuncheon, Gangwon [36]. Afterward, Yi et al. found 5 (7.1%) human anaplasmosis cases among 70 Koreans who underwent bone marrow examination due to fever and hemocytopenia. The five anaplasmosis cases were confirmed by PCR, and one of them revealed morulae in the PBS [37]. The detection rate of morulae is known to be 25–75% in the first week of the disease [5, 38]. In our study, one of the anaplasmosis cases was also diagnosed by morulae found within granulocytes in the PBS. The patient presented fever, dizziness, and myalgia, and laboratory results showed pancytopenia and increased CRP, AST, ALT, LDH, and BUN. The leptangamushi antibody test was positive for *O*. *tsutsugamushi*. The IFA assays were all negative, but the PCR was positive for *A*. *phagocytophilum*. The patient samples were taken on the fifth day after symptom onset. In a previous case report, the *A*. *phagocytophilum* IFA result on day 5 was negative and the PCR was positive, and the IFA titer began to increase on day 10, whereas the PCR turned negative [39]. The results on day 5 were consistent with our case, but the follow-up sample of this case was not included in this study. Considering the negative *O*. *tsutsugamushi* PCR result, it can be inferred that the positive *O*. *tsutsugamushi* antibody was caused by the possibility of infection in the past.

In this study, 85.2% of the *A*. *phagocytophilum* seropositive group harbored antibodies to *Borrelia*, indicating presumptive evidence of sharing the same vector. However, only 17.3% of the *A*. *phagocytophilum* seropositive group harbored antibodies to *E*. *chaffeensis*, and the overall seropositivity to *E*. *chaffeensis* and titers were relatively low to other pathogens. Only five patients exhibited high titers of *E*. *chaffeensis*. A previous Korean study showed that 1.0% of 1,618 ticks (*H*. *longicornis*, *I*. *persulcatus*, *and I*. *nipponensis*) were *E*. *chaffeensis* positive via PCR [40–44], and they suggested the distribution of *E*. *chaffeensis* throughout South Korea [25]. Although the seroprevalence of *E*. *chaffeensis* may be low in Korea, it is necessary to be cautious in cases with a high titer of *E*. *chaffeensis*, which may indicate a high burden of tick-borne disease. The presence of the causative agents and potential tick vectors with the capacity to bite humans suggests that the serological data reflect a previously unrecognized but emerging problem in South Korea.

In this study, 87.9% of the *B*. *henselae* antibody-positive cases harbored multiple antibodies to vector-borne pathogens. A relatively low titer of *B*. *henselae* was observed in the OFD group, whereas a high titer was noticed in the ST group. In Korea, serologic and molecular evidence for *B*. *henselae* and *B*. *quintana* was observed in ticks and small animals [41, 44, 45]. According to a previous study, *Bartonella* DNA was isolated from *H*. *longicornis*, *H*. *flava*, *I*. *persulcatus*, and *I*. *nipponensis* [46]. Human infections of *B*. *henselae* and *B*. *quintana* were also described [47–49]. The precise incidence of bartonellosis in Korea has not yet been investigated; however, those reports, including this study, suggest that the burden of bartonellosis in Korea could be higher than expected.

We found the possibility of the coinfection of multiple vector-borne pathogens in febrile illness patients, demonstrating the seropositivity of those pathogens. Primarily, the high titers of antibodies to multiple pathogens support the possibility of co-existence. Follow-up cases also strengthened the possibility of coinfection of ST and other VBDs. Four of the five follow-up ST patients were already seropositive to other vector-borne pathogens, suggesting previous exposure. A previous study of 91 individuals who recovered from ST, the follow-up IgM, IgG, and total Ig positivity rates for 13 years were 37.4% (34/91), 22.0% (20/91), and 76.9% (70/91), respectively [50]. Almost all patients with ST had a frequent outdoor activities history, suggesting that they might be persistently exposed to the risk of tick or mite bites. In follow-up evaluation, the appearance of new antibodies or an increase in the pre-existing antibody titers was detected. Such changes support the possibility of a coinfection of *O*. *tsutsugamushi* and other vector-borne pathogens.

Meanwhile, the positive reaction in IFA assay may be due to the cross-reactive immune responses to vector-borne pathogen-related antigens. They are typically group-specific, although perhaps not species-specific. Previous reports announced that antibodies reactive against *E*. *chaffeensis* or *A*. *phagocytophilum* could react with other species, impeding epidemiologic distinction between the infections [51]. In our data, a cross-reactive effect might exist in *E*. *chaffeensis*, considering that its average titer was very low (1+). The possibility of a cross-reaction between the antibodies of those pathogens needs to be further evaluated.

The diagnosis of vector-borne infection generally relies on serologic tests using indirect immunofluorescence assays (IFAs) showing at least a 4-fold increase in the antibody titers between paired sera [52, 53]. However, the need for the paired serum samples to be taken over a specific period is the most crucial factor that explains the low effectiveness of IFA tests during the acute phase of the disease. Additionally, to perform the IFA test, conditions such as fluorescent microscopes, dark rooms, and trained laboratory personnel are required. Furthermore, serologic evaluations for vector-borne pathogens other than ST are not usually performed in general laboratories because it is not yet permitted by the Korean Ministry of Food and Drug Safety for clinical diagnosis. Real-time PCR has also been proposed for the early diagnosis of vector-borne infection [54], but buffy coat samples are needed, which require technical expertise for their preparation. The clinical sensitivity of a real-time PCR using serum samples is insufficient and is not commercially available [55].

In the clinical field in South Korea, there is little choice in choosing laboratory tests to diagnose VBDs. IFA assays and PCRs for most vector-borne pathogens are unavailable for routine tests and can only be used for research. Therefore, most clinical diagnoses are restricted to the pathogens only available for routine antibody testing, such as *O*. *tsutsugamushi*. Therefore, diagnosing VBDs usually depends on the physician’s experience and clinical evidence. With this study, we want to show the possibility of the co-infection of other VBD with *O*. *tsutsugamushi* and provoke the recognition of the need for further laboratory evaluation. We suggest that when a patient is suspected of VBD, the IFA tests should be performed for the major pathogens. As shown in our data, the positive rate of PCR is relatively low. Previous reports announced that PCR-negative results do not exclude infection, as the presence in the blood of some vector-borne pathogens can be temporal and transient [56]. In this study, the patient clinically diagnosed with anaplasmosis showed a positive PCR and a negative IFA for *Anaplasma*. Likewise, molecular diagnosis can be helpful in the acute infection stage when antibodies are at low titers or negative.

Our work had several limitations. First, the titers by the directly diluted test samples were not taken. We indirectly compared the sample fluorescence with the diluted positive control. However, by only counting the fluorescence stronger than that of the 1:256 diluted positive control as positive, the positive result has enough value to suggest the seropositivity. Second, the baseline seroprevalence in healthy controls was not evaluated. A Korean study about ST reported no seroprevalence of IgG with a cutoff value of ≥1:256 among 216 health checkup personnel [50]. The baseline seroprevalence of the other four vector-borne pathogens is unknown in this geographical region; therefore, a further study of antibody and titer analysis for the four vector-borne pathogens is required. Third, along with the positive IgG antibody result, we did not test the IgM antibody, which could provide further information to distinguish between present and past infections. Fourth, cross-reactions among the pathogens should be excluded using assays such as Western blot. Last, we did not evaluate any samples other than blood samples. Molecular studies using tissue samples, such as eschar, may aid in the determination of the causative agents [57].

Here, we found that the co-existence of vector-borne pathogens in ST and other febrile illnesses may be underestimated. Coinfections should be considered in actual clinical practice and also in the laboratory. Acute febrile illness and manifestations suggestive of vector-borne infection must be recognized and further explored in order to determine the appropriate treatment. Further evaluation methods, such as IFA antibody testing and PCR, are needed to be introduced for routine laboratory work.

## Supporting information

S1 Fig Representative curves of the real-time PCR commercial kits for vector-borne pathogens used in this study. (A) Positive controls included in the kits. Real-time PCR assays for four other tick-borne pathogens were all negative (B), but only one case showed a positive amplification curve to the *Anaplasma* target (C). This positive reaction for *A*. *phagocytophilum* was confirmed by additional sequencing, and the sequence was deposited to GenBank (Accession no. OQ750553).
(PDF)

S1 Table Primer sequences used for confirmation of positive reaction in real-time PCR assay. (DOCX)
