# Supplementary material for: Under-diagnosis of vector-borne diseases among individuals suspected of having Scrub Typhus in South Korea
Source: PLoS One. 2023 Jun 2;18(6):e0286631. doi: 10.1371/journal.pone.0286631 (PMC10237470; doi:10.1371/journal.pone.0286631)
Supplement: S1 Table — (DOCX) [file pone.0286631.s002.docx]

Supplemental Table 1. Primer sequences used for confirmation of positive reaction in real-time PCR assay

| Target | Primer | Primer sequences (5' to 3') | Product size | Reference |
| --- | --- | --- | --- | --- |
| *Orientia tsutsugamushi* | 56BO_144F | YGYAGAATCTRCTCGCTTGG | 1250bP | [14] |
|  | 56BO_1395F | AGCTAMCCCTRCACCAABAC |  |  |
| *Anaplasma phagocytophilum* | ANK_F1 | GAA GAA ATT ACA ACT CCT GAA G | 705bP | [18] |
|  | ANK_R1 | CAG CCA GAT GCA GTA ACG TG |  |  |
| *Bartonella spp.* | PBH3 | CCA AGT GCT ACA TAA CCA TC | 1723bp | [19] |
|  | PBH4 | CGG GTT GTT ATT GCT CTT AC |  |  |
| *Borrelia spp.* | Bor-16F | TGC GTC TTA AGC ATG CAA GT | 1344bp | [20] |
|  | Bor-1306R | GTA CAA GGC CCG AGA ACG TA |  |  |
| *Ehrlichia chaffeensis* | ECH50-68 | CTT CTC TAC CTG GAG TAT C | 620bp | [21] |
|  | ECH669-649 | GCT TAT AGA GTA GCT TAA ACC |  |  |
